# Desalted *Salicornia europaea* powder and its active constituent, *trans*-ferulic acid, exert anti-obesity effects by suppressing adipogenic-related factors

**DOI:** 10.1080/13880209.2018.1436073

**Published:** 2018-03-09

**Authors:** Md. Mahbubur Rahman, Myung-Jin Kim, Jin-Hyoung Kim, Sok-Ho Kim, Hyeon-Kyu Go, Mee-Hyang Kweon, Do-Hyung Kim

**Affiliations:** a Research Center, KNOTUS Co. Ltd, Guri-Si, Gyeonggi-Do, Republic of Korea;; b College of Veterinary Medicine and Institute of Veterinary Science, Kangwon National University, Chuncheon, Gangwon, Republic of Korea;; c Department of Biofood research, Knotus Life Science Inc, Jeongeup, Republic of Korea;; d Research Center, Phyto Corporation, Seoul, Republic of Korea

**Keywords:** Desaltation, adipogenesis, 3T3L-1 cells, abdominal fat mass

## Abstract

**Context:***Salicornia europaea* (Amaranthaceae) (SE) has been shown to reduce obesity, but it remains a problem as a food supplement because of its high salt content (25–35% NaCl).

**Objectives:** This study investigated the anti-obesity effects and mechanism of action of desalted SE powder (DSP).

**Materials and methods:** Sprague–Dawley rats (*n* = 50) were divided into a normal control group (NC), a high-fat diet (HFD)-induced obesity control group (HFD), and HFD groups co-administered DSP (250 and 500 mg/kg) or *Garcinia cambogia* (Clusiaceae) extract (GE, 200 mg/kg, standard control) orally each day for 12 weeks.

**Results:** The body weight was significantly reduced by co-administration of DSP (596.51 ± 19.84 kg, 4.60% and 562.08 ± 9.74 kg, 10.10%, respectively) and GE (576.00 ± 11.29 kg, 7.88%) relative to the HFD group (625.25 ± 14.02 kg) and was accompanied by reduced abdominal fat mass, and serum lipid levels, with no effects on feed intake. To find the underlying mechanism of the anti-obesity effects, *trans*-ferulic acid (TFA) was identified as the main ingredient and investigated with regard to whether it attenuated adipogenesity in 3T3L-1 cells. DSP-derived TFA suppressed adipocyte differentiation and accumulation of intracellular lipids. TFA also down-regulated the adipogenesis-related gene expression of sterol regulatory element-binding protein 1, peroxisome proliferator-activated receptor γ, CCAAT/enhancer binding protein-α and fatty acid synthase.

**Conclusions:** These findings suggest that DSP may be considered for use as a food supplement intent of controlling obesity through its antiobesity and antiadipogenic properties.

## Introduction

Obesity is associated with a number of health disorders, including dyslipidemia, diabetes, cardiovascular disease, gall bladder disease, cerebro-vascular diseases, obstructive sleep apnea, menstrual irregularities and certain types of cancer (Haslam and James 2005). It results from an imbalance between energy intake and expenditure, which may lead to a pathological growth of adipocytes (Yuan and Piao 2011). Obesity rates are rapidly increasing and reaching alarming levels worldwide due to habits in modern lifestyles such as physical inactivity, sitting in chairs for work, watching television and high-caloric intake (Haslam and James 2005; Malik et al. 2013; Abid et al. 2016).

Adipogenesis and adipocyte differentiation are important for obesity and involve a complex sequence of changes in gene expression and lipid storage (Yuan and Piao 2011). Controlling adipogenisis or obesity is essential for preventing complications and improving the health of obese patients. Currently, the drugs available for the treatment of obesity promise short-term benefits, but the obesity returns when the medications are discontinued. Moreover, these medications have a number of side effects. Thus, recent efforts for the complementary treatment of obesity have focused on functional foods and their bioactive compounds (Hasani-Ranjbar et al. 2013; Ko et al. 2015; Pichiah and Cha 2015; Abid et al. 2016; Guo et al. 2016).

*Salicornia europaea* (Amaranthaceae) (SE) known as jointed glasswort is native to the Mediterranean but is also abundant in Korea and Japan. It is commonly consumed in salads or as an ingredient in various recipes (Fita et al. 2015; Panth et al. 2016). It is also known as *Salicornia herbacae*, and its local name in Korea is hamcho. It mainly grows in high-salt coastal marshes (Panth et al. 2016). It has recently emerged as commercial edible halophyte in the agriculture sector and can be cultivated by using sea water irrigation without any fertilizers or pesticides (Fita et al. 2015). Carbohydrates, proteins, minerals, oils, phenolic compounds, flavonoids, sterols, saponins, alkaloids and tannins have been identified in SE (Kim et al. 2009; Cho et al. 2016). It has antioxidative, immune-modulatory, hepatoprotective, anti-inflammatory, antilipidemic, antihypertensive, antidiabetic, antimicrobial and anticancer activities (Kim et al. 2009; Cho et al. 2016). Normal SE powder (NSP) contains high salt content (25–35% NaCl) and dietary fibre, which have been proven to limit body weight gain and obesity in previous experiments (Lee et al. 2012; Pichiah and Cha 2015; Kim et al. 2016). NSP and an amount of NaCl equal to that in NSP were co-administered in separate groups with a diet-induced obesity model (Pichiah and Cha 2015) and high salt-induced hypertension model (Panth et al. 2016). NSP could be used as a salt substitute to prevent weight gain (Pichiah and Cha 2015), vascular dysfunction and hypertension (Panth et al. 2016). Desalted SE powder (DSP) could be more attractive and a better alternative as a functional food compared to NSP to combat obesity.

*Garcinia cambogia* (Clusiaceae) is a well-known and popular hypolipidemic herbal plant (Marquez et al. 2012; Vasques et al. 2014). It is readily available in markets as a food supplement and contains hydroxycitric acid (HCA) as its active ingredient. This study investigates the attenuating effects of DSP on body weight gain and visceral adiposity (total abdominal fat, visceral abdominal fat and subcutaneous abdominal fat) in an obese rat model in comparison with *Garcinia cambogia*. To determine the underlying mechanism involved in the anti-obesity effect, *trans*-ferulic acid (TFA) was identified by HPLC analysis as the most abundant component in DSP, and it was also investigated in regard to whether DSP-derived TFA could attenuate adipogenisity and adipogenic-related factors in mouse 3T3L-1adipocytes.

## Materials and methods

### Preparation of desalted salicornia power (DSP) and analysis of DSP constituents by HPLC

Fresh leaves, branches and stems of SE (20.0 kg) were collected from the western seashore of South Korea at the end of August 2015. The samples were verified by Prof. Soon-Ae Yoo from the Department of Biology and Medicinal Science, Pai Chai University (Daejon, Korea). A voucher specimen was deposited in a freezer located in the R & D center of the Phyto Corporation (Seoul National University, South Korea). The samples were cleaned with tap water and lyophilized using a freeze-dryer (EYELA FDV-2200, Tokyo, Japan). The lyophilized SE was ground using a blender (EBR98045, Stockholm, Sweden), and the powdered SE (3.6 kg) was desalted by water extraction and centrifuged (Kim et al. 2016).

The pellet obtained from the centrifugation was lyophilized and ground into a fine powder with less than 150 mesh size using an ultra-centrifugal mill (ZM200, Hann, Germany). The resulting DSP (2.4 kg) comprised 74.3% dietary fibre, 9.1% protein, 4.3% fat and 0.71% sodium (Table 1). A nutritional analysis of the DSP was conducted and certified by the Korea Health Supplement Association (Bundang-gu, Gyeonggi-do, Republic of Korea). Briefly, the total carbohydrate content was calculated as the sum of anhydrosugars, which was determined using the phenol-sulfuric acid method (Dubois et al. 1956). The total protein content was determined using the protein digestion/micro-Kieldahl method (Willis et al. 1996), while the sodium content and total content of dietary fibre were analyzed by inductively coupled plasma (ICP) and enzymatic-gravimetric methods, respectively. The caloric content and other minor components were determined according to the methods of the Association of Official Analytical Chemists (AOAC) (Prosky et al. 1985).

**Table 1.** Chemical composition of desalted *Salicornia europaea* powder (DSP).

Analytical result	
Calorie	224.89 Kcal/100g
Dietary fibre	74.27%
Crude protein	9.13%
Crude fat	4.36%
Ash	6.23%
Sodium	0.71%
Saccharide	ND
Saturated fatty acid	0.45%
Trans fatty acid	>0.01%
Cholesterol	ND

To analyze the major low-molecular-weight compounds in DSP, an aliquot of DSP (10 g) was suspended in 100 mL of distilled water containing digesting enzymes such as amylase and protease at 37 °C for 6 h, which was then centrifuged at 10,000 *g* for 25 min. The resulting supernatant was concentrated at reduced pressured to obtain an extract (DSP-EW, 950 mg). The enzyme-digested water extracts (DSP-EW, 100 mg) was extracted with methanol, and the methanol-soluble DSP-EW was used to prepare an HPLC sample (20 mg/mL). HPLC experiments were performed using an Agilent HPLC instrument (Agilent Technologies, Santa Clara, CA, USA) with a 1260 quaternary pump, 1260 ALS auto sampler, 1260 DAD diode-array detector, and 1260 TCC thermostated column compartment. The methanol-soluble DSP-EW sample (10 mg/mL, 3 μL) was injected into a Zorbax Eclipse Plus C18 analytical column (4.6 × 150 mm, 3.5 μm, Agilent) and analyzed with a gradient eluent of methanol and water with a flow rate of 1.0 mL/min and column temperature of 25 °C. HPLC profiles and the UV spectrum were recorded using an Agilent UV detector (1260 DAD, 190–400 nm, 20 nm step) at 300 nm/reference 360 nm. UV spectra of the major compound **1** (MeOH, Agilent, 1200 DAD, 190–400 nm, 20 nm step) showed λ-maxima at 218–220, 240, 285–290 and 325 nm, which are characteristic of phenolic acids (Markham 1982).

**Figure 1.**
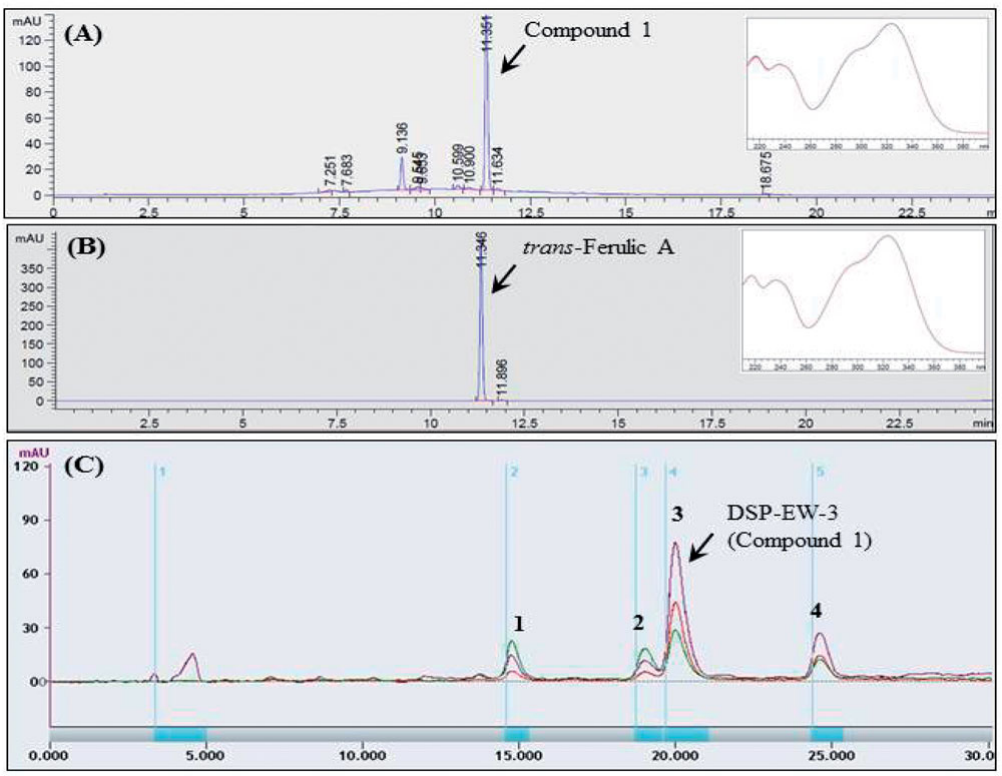
Analytical and preparative HPLC profiles of desalted *Salicornia europaea* L. powder (DSP). (A) Analytical HPLC profile of DSP-EW; (B) analytical HPLC profile of authentic *trans*-ferulic acid; (C) Multiple preparative HPLC profile of DSP-EW; (1) caffeic acid; (2) *p*-coumaric acid; (3) *trans*-ferulic acid; (4) isorhamnetin-3-β-D-glucoside.

By comparing the HPLC retention time and the λ-maxima of UV absorption of the authentic phenolic compounds (Sigma Co), the major compound **1** was identified as *trans*-ferulic acid (TFA) (Figure 1(A,B)). The methanol-soluble DSP-EW (800 mg/mL) was purified by a multiple preparative HPLC (LC-Forte, YMC, Kyoto, Japan) equipped with a Triart C18 prep column (20 × 250 mm, 5 μm, YMC, Kyoto, Japan) and a gradient eluent of MeOH and water to give pure TFA (DSP-EW-3, Compound 1, 128 mg) of RT: 19.5–21.2 min, and other minor peak compounds were also identified as caffeic acid, *p*-coumaric acid and isorhamnetin-3-β-d-glucoside (Figure 1(C)). For further identification, molecular weight of the purified DSP-EW-3 (Compound 1) was determined using ESI-MS. DSP-EW-3 dissolved in methanol was ionized by spray ionization (150 ∼ 2000 *m/z*) and ESI-MS spectra were obtained on a LC-ESI mass spectrometer (Thermo Finnigan LTQ, X-caliber software, version 2.1.0; Thermo Finnigan, San Jose, CA, USA) with both positive and negative modes.

**Figure 2.**
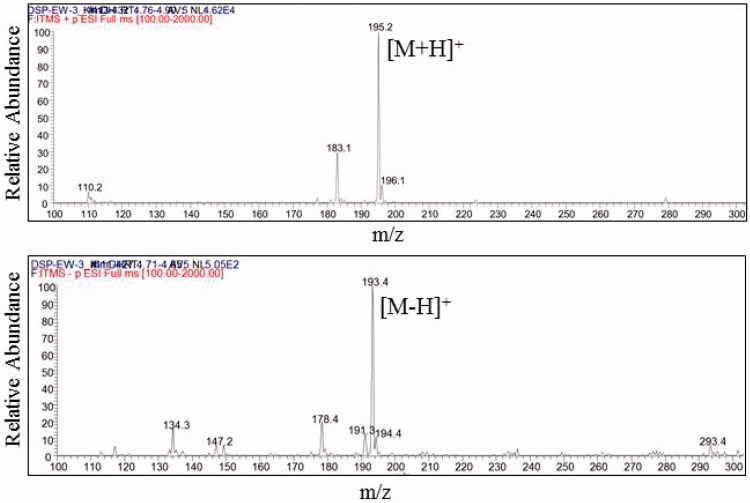
ESI-MS spectra of DSP-EW-3 (compound 1) (*top*, positive; *bottom*, negative).

The ESI-MS results, *m/z* 195.2 [M + H] and m/z 193.4 [M-H], showed that the molecular weight of compound **1**, DSP-EW-3 was same as that of *trans*-ferulic acid (MW, 194, C10H10O4) (Figure 2). Readily available Garcinia cambogia extract (GE) (Pure GARCINIA Cambogia^®^, Fusion Diet Systems Inc., Sandy, UT) containing 60% hydroxycitric acid (HCA) was used as a positive control (200 mg/kg). Higher doses of simple DSP were selected (250, 500 mg/kg) compared to GE.

### Animals and experimental design

Male white Sprague–Dawley rats (Orient Bio, Gapyeong, Gyeonggi-do, Korea) were used for this study. The rats were housed in a controlled environment with temperature of 23 ± 2 °C and humidity of 50 ± 5% with 12 h light/dark cycle. Food and water were available *ad libitum* before starting the experiment. After a week of adaptive feeding, the average body weight was 222 ± 2 g. Obesity was induced using a 60% high-fat diet (HFD) for 12 weeks, while a normal control group (NC) was given a regular diet. The rats (*n* = 50) were divided equally in to five groups: a normal control (NC) treated with saline in a matched volume, an HFD group (obese rat model fed an HFD, an HFD + DSP-250 group (co-administered DSP-250 mg/kg orally daily with HFD), an HFD + DSP-500 group (co-administered DSP-500 mg/kg daily orally), and an HFD + GE-200 group (co-administered GE, 200 mg/kg daily orally). DSP and GE were mixed with distilled water and administered (10 mL/kg body weight) freshly by oral gavage. All experimental protocols were approved by the animal care and use committee of KNOTUS Co. Ltd. Korea (Certificate number: IACUC 16-KE-097).

### Measurement of abdominal fat in live animals

Non-invasive abdominal visceral adiposity parameters such as total abdominal fat (TAF), visceral abdominal fat (VAF), and subcutaneous abdominal fat (SAF) volume were measured in live animals using three-dimensional micro-computed tomography (micro-CT). Micro-CT measurements were performed on a Scanco Viva CT 80 (Scanco Medical AG, Bassersdorf, Switzerland) at 12 weeks according to the manufacturer's directions. For scanning, rats were anesthetized using 1% isoflurane inhalation and positioned on their back with the face up and head toward the front. Both hind limbs were extended and fixed to a specimen holder with an angle of 90° between the femur and spine with both legs fully extended.

To quantify visceral and subcutaneous fat, the area between the proximal end of lumbar vertebra L1 and the distal end of L6 was scanned at an isotropic voxel size of 18 μm (70 kVp energy, 114 μA intensity, 31.9 mm FOV/diameter, 200 ms integration time). Selection of the scan energy and voxel size (scanning increment) was based on optimizing the scanning time and tissue detail and minimizing exposure to radiation according to the company's directions. Each parameter was analyzed from 3D reconstructions of the micro-CT scans generated by the provided imaging software (Scanco Medical, Bassersdorf, Switzerland).

### Measurement of serum biochemical parameters

Approximately 1 mL of blood was collected from the jugular vein of the animals using vacutainer tubes containing a clot activator at 8 weeks and 12 weeks after inducing obesity. Coagulation was allowed to occur at room temperature for approximately 15 to 20 min, and then the serum was separated by centrifugation at 3000 rpm for 10 min and stored at −20 °C until analysis. Serum triglyceride (TG), total cholesterol (TC), high-density lipoprotein (HDL), and low density lipoprotein (LDL) levels were measured with a Hitachi 7180 instrument (Hitachi, Tokyo, Japan). The serum level of very low density lipoprotein cholesterol (VLDL) was calculated using the formula: VLDL = TG/5 (Friedewald et al. 1972; Rahman et al. 2017). The atherogenic index (AI) is a useful tool for assessing cardiovascular disorders related to serum lipids and was measured using the TC/HDL ratio (Grover et al. 1999; Rahman et al. 2017).

### Cell culture and adipocyte differentiation

Mouse 3T3-L1 pre-adipocytes were cultured at 37 °C and 95% humidity with a 5% CO_2_ atmosphere in Dulbecco’s modified eagle medium (DMEM) as the culture medium along with 10% foetal bovine serum (FBS), 2 mL of l-glutamine, 50 U/mL of penicillin and 50 μg/mL of streptomycin, which was incubated for 3 days for confluence. At this point (day 0), differentiation medium (DM) (DMEM containing 10% FBS, 2 mL of l-glutamine, 50 U/mL of penicillin, 50 μg/mL of streptomycin, 0.5 mM IBMX, 1 µM dexamethasone and 1.7 µM insulin) was added for 6 days and was changed at 3 day intervals. On the sixth day, the differentiation medium was replaced by induction medium (DMEM containing 10% FBS, 2 mL of l-glutamine, 50 U/mL penicillin, 50 μg/mL of streptomycin, 1.7 µM insulin). The NC medium was treated with only culture medium and differentiation and induction medium (DMI). The rest of the groups were treated with different concentrations of TFA along with differentiation and induction medium (DMI +10, 50, 100, 500 μM TFA) between days 0 and 9. The cytotoxicity effect of TFA on 3T3-L1 cells was evaluated by MTT assay. The concentrations of TFA were 0–500 μM and no cellular toxicity was observed within this range.

### Triglyceride assay and oil red O staining

To investigate the anti-adipogenic effects of DSP, 3T3-L1 pre-adipocytes were treated with DSP-purified TFA for 9 days (days 0 to 9). The effect of TFA on the induction of terminal differentiation markers was evaluated at the end of differentiation (day 9). To determine the cellular lipid content, cells were collected and lysed in lysis buffer (25 mM sucrose, 20 mM Tris-HCl, 1 mM EDTA and 1 mM EGTA). Cellular triglyceride contents were measured using a commercial triglyceride quantification kit (Abcam, MA) according to the manufacturer’s instructions.

Lipid accumulation in the cytoplasm was analyzed using an Oil Red O staining kit according to the manufacturer’s suggested protocol (Lifeline Cell Technology, Carlsbad, CA). Stained lipid droplets in cells were viewed and imaged with an Observer A1 microscope (Carl Zeiss, Jena, Germany) at 100 × magnification. The absorbance values of eluted Oil Red O solution were measured in the adipocyte reflex lipid droplet accumulation in the cytoplasm, and so was the neutral lipid content. After taking a photograph, the stained culture dye retained in the cells was eluted with isopropanol and quantified at an absorbance rate of 540 nm using a multiplate reader spectrophotometer (BioTek Instruments, VT).

### RNA isolation and real time RT-PCR

The effect of DSP on the adipogenesis-related cellular expression of transcription factors SREBP1, PPAR-γ, C/EBPα and FAS was investigated in 3T3L-1 cell lysates using RT-PCR. Total cellular RNA was isolated from 3T3-L1 adipocytes using an easy BLUE kit (iNtRON, INC., Daejeon, Korea) and employed in real-time RT-PCR using a CFX96TM real-time PCR detection system (Bio-Rad Laboratories, Hercules, CA). Reverse transcription of total RNA was conducted with high-capacity cDNA reverse transcription kits (Applied Biosystems, CA), and the reaction mixture contained 2 μL of template cDNA, 10 μL of 2 × SYBR Primix Ex Taq and 200 nM primers in a final volume of 20 μL.

The reactions were denatured at 95 °C for 30 s and then subjected to 45 cycles of 95 °C for 5 s and 60 °C for 20 s. When the reaction cycle was completed, the temperature was increased from 65 °C to 95 °C at a rate of 0.2 °C/15 s. The fluorescence was measured every 5 s to construct a melting curve. A control sample that contained no template DNA was run with each assay, and all determinations were performed at least in duplicate to ensure reproducibility. The authenticity of the amplified product was determined by melting curve analysis. All data were analyzed using the analysis software Bio-Rad CFX Manager Version 2.1 (Bio-Rad Laboratories). The target cDNA was amplified using the sense primer and antisense primers as shown in Table 2.

**Table 2.** Primer sequences utilized for real time RT-PCR evaluation of gene expression.

Gene	Primer sequence	
	Sense	Anti-sense
C/EBPα	5′-AAA CAA CGC AAC GTG GAG A-3′	5′-GCG GTC ATT GTC ACT GGT C-3′
FAS	5′-GCT GCT GTT GGA AGT CAG C-3′	5′-AGT GTT CGT TCC TCG GCG TG-3′
PPARγ	5′-CAA GCC CTT TAC CAC AGT TGA-3′	5′-CAG GTT CTA CTT TGA TCG CAC TT-3′
SREBP-1	5′-AAC CAG AAG CTC AAG CAG GA-3′	5′-TTT CAT GCC CTC CAT AGA CA-3′
GAPDH	5′- CAT GGC CTT CCG TGT TCC TA-3′	5′- CCT GCT TCA CCA CCT TCT TGA T-3′

### Statistical analysis

The results were assumed to be normally distributed and analyzed by parametric multiple comparison procedures and one-way analysis of variance (ANOVA). When the results of ANOVA were significant, Dunnett’s multiple comparison test was applied. The statistical analyses were performed with Prism 5.03 (GraphPad Software Inc., San Diego, CA), and the significance level was set at *p* < 0.05.

## Results

### Effects of DSP on body weight, feed intake and visceral adiposity

**Figure 3.**
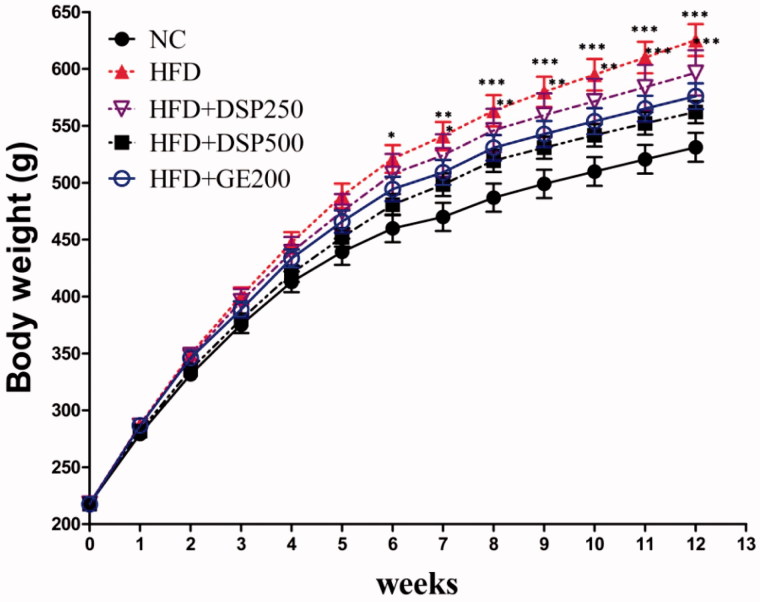
Effect of co-administration of DSP on body weight in high fat induced obese rat. NC: normal control; HFD: high-fat diet induced obese group; HFD + DSP-250, rat supplemented HFD and desalted *S. europea* powder at dose 250 mg/kg; HFD + DSP-500, rat supplemented 500 mg/kg DSP; HFD + GE-200, rat supplemented 200 mg/kg *Garcinia cambogea* extract. The data are reported as the mean ± SD (*n* = 10). *p < 0.05, **p < 0.01, ***p < 0.001, Bonferroni *post hoc* test following two-way ANOVA versus the NC group.

Sharply elevated body weight was found in rats after HFD supplementation in the whole experiment. However, concomitant DSP supplementation reduced the occurrence of elevated body weight (Figure 3) without alteration of feed intake (Table 3) compared to the HFD group. However, significantly reduced feed intake (*p* < 0.05) was observed in all HFD groups compared to the NC group. It is possible that the rats have little tendency to intake an HFD. At the end of the experiment, the final body weight was lower in the HFD + DSP-250, HFD + DSP-500 (*p* < 0.001) and HFD + GE-200 (*p* < 0.001) groups than in the obese HFD group (Figure 3).

**Table 3.** Effect of DSP supplementation on average feed intake per day and serum lipid profiles, AST, ALT levels of rat.

	NC	HFD	HFD + DSP-250	HFD + DSP-500	HFD + GE-200
Feed intake (g/day)	24.27 ± 0.46	16.84.9 ± 0.38*	17.49 ± 0.32*	17.03 ± 0.31*	16.31 ± 0.21*
TC (mg/dl)	71.3 ± 2.9	151.9 ± 3.5**	144.8 ± 5.1*	131.6 ± 5.6**##	137.6 ± 4.4**#
TG (mg/dl)	117 ± 7	171.2 ± 8.5**	149.7 ± 4.6**#	130.5 ± 6.6*##	135.7 ± 5.8*##
HDL (mg/dl)	28.6 ± 0.7	29.1 ± 0.8	29.3 ± 1.2	30.7 ± 1.2	29.7 ± 0.7
LDL (mg/dl)	8.9 ± 0.9	16.6 ± 0.9***	16.0 ± 1.0**	14.9 ± 0.8*#	15.8 ± 0.6*
VLDL (mg/dl)	17.6 ± 1.5	34.2 ± 1.7**	29.9 ± 0.9*##	26.1 ± 1.3###	27.1 ± 1.2###
AI	2.49 ± 0.05	5.22 ± 0.07***	4.97 ± 0.10***#	4.30 ± 0.08**###	4.62 ± 0.08**###
AST(U/l)	129.1 ± 7.2	181.8 ± 12.3**	167.9 ± 9.9*	149.0 ± 8.0#	154.3 ± 9.5
ALT(U/l))	60.1 ± 3.8	137.6 ± 4.8***	123.7 ± 4 **#	110.1 ± 3.9*##	118.4 ± 4.6**#

NC: normal control; HFD: high-fat diet induced obese group; HFD + DSP-250: rat supplemented HFD and desalted *S. europea* powder at dose 250 mg/kg; HFD + DSP-500: rat supplemented 500 mg/kg DSP; HFD + GE-200: rat supplemented 200 mg/kg *Garcinia cambogea* extract. TG: triglyceride; TC: total cholesterol; HDL: high-density lipoprotein; LDL: low-density lipoprotein; VLDL: very low-density lipoprotein; AI: atherogenic index; AST: aspartate aminotransferase; ALT: alanine aminotransferase. The data are reported as the mean ± SD (*n* = 10). *p < 0.05, **p < 0.01, ***p < 0.001, analyzed by parametric multiple comparison procedures, One-way ANOVA test. When the result of ANOVA was significant, and Dunnett’s multiple comparison test was applied *versus* the NC group; ^#^*p <* 0.05; ^##^*p <* 0.01; and ^###^*p <* 0.001, versus high-fat diet supplemented group (HFD).

**Figure 4.**
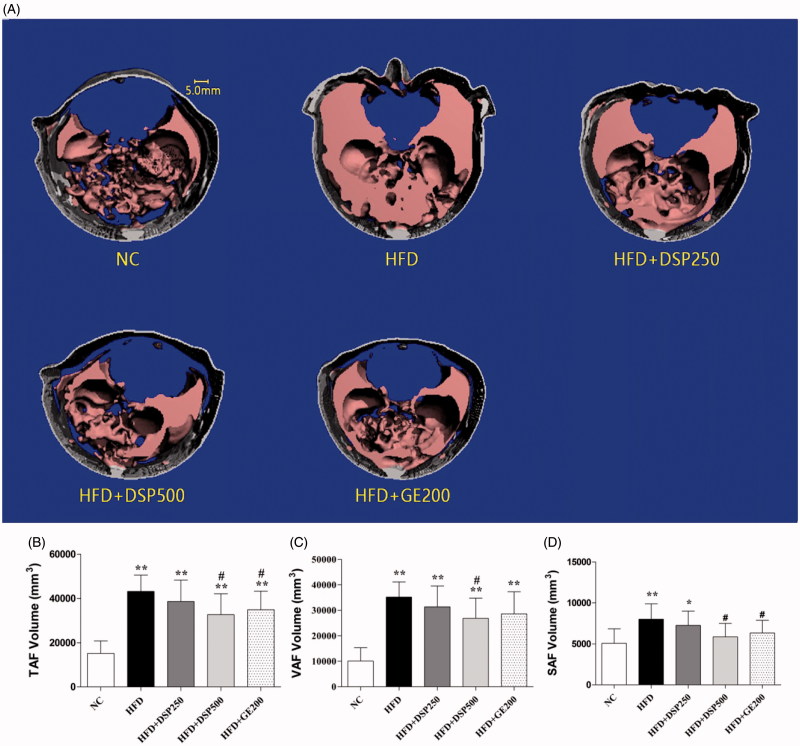
Effect of co-administration of DSP on body weight in high fat induced obese rat. NC: normal control; HFD: high-fat diet induced obese group; HFD + DSP-250, rat supplemented HFD and desalted *S. europea* powder at dose 250 mg/kg; HFD + DSP-500, rat supplemented 500 mg/kg DSP; HFD + GE-200, rat supplemented 200 mg/kg Garcinia cambogea extract. (A) 3-D micro-CT images of abdominal adiposity. (B–D) numerical presentation of abdominal adiposity. TAF: total abdominal fat; VAF: visceral abdominal fat, and SAF: subcutaneous abdominal fat volume. The data are reported as the mean ± SD (*n* = 10). *p < 0.05, **p < 0.01, ***p < 0.001, analyzed by parametric multiple comparison procedures, One-way ANOVA test. When the result of ANOVA was significant, and Dunnett’s multiple comparison test was applied versus the NC group; #*p* < 0.05; ##*p* < 0.01; and ###*p* < 0.001, versus high-fat diet supplemented group (HFD).

Micro-CT scans showed that the abdominal fat contents of TAF, VAF and SAF were all increased in the HFD-induced obese rats compared with the normal-diet rats (Figure 4). However, concomitant DSP administrations reduced the amount of excess fat content that occurred. TAF, VAF and SAF were reduced by 30% and 20%, respectively. Interestingly, DSP was more effective at reducing fat content than GE.

### Effect of DSP on lipid profiles and atherogenic index

The serum levels of TC, LDL, VLDL, TG and the atherogenic index sharply increased in the HFD group (1.13, 0.90, 0.94, 0.94 and 1.10-fold, respectively) compared with the normal group. No significant alteration was observed in the serum levels of HDL. However, these conditions were improved after 12 weeks of DSP supplementation. The most significant improvement was found in the HFD + DSP-500 group (Table 3).

### Effect of DSP-purified TFA on cell toxicity and intracellular lipid accumulation in adipocytes and on adipogenic-related gene and protein expression

**Figure 5.**
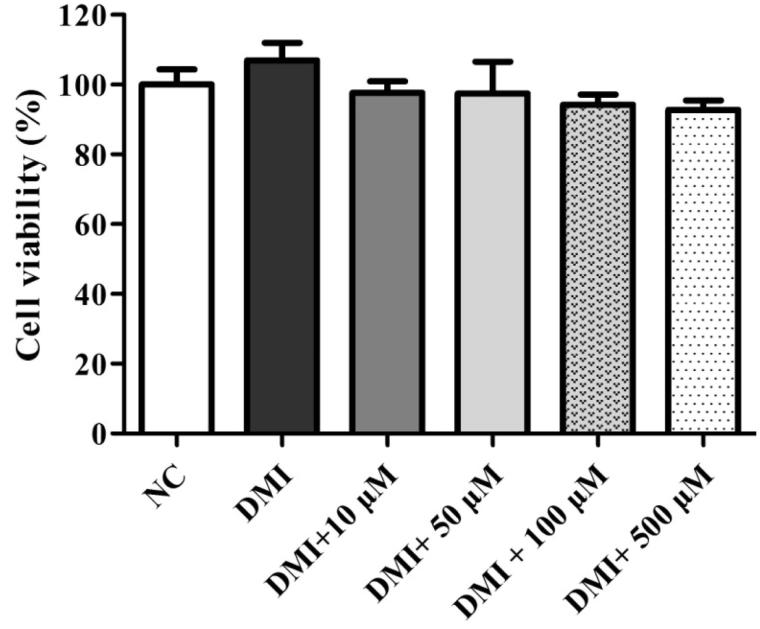
DSP-derived trans-ferulic acid on cell viability in 3T3-L1 cells. The data are reported as the mean ± SD (*n* = 10). Analyzed by Bonferroni *post hoc* test following one-way ANOVA versus the NC group. But there was no significant change was observed either versus normal control (NC) or versus DMI group.

**Figure 6.**
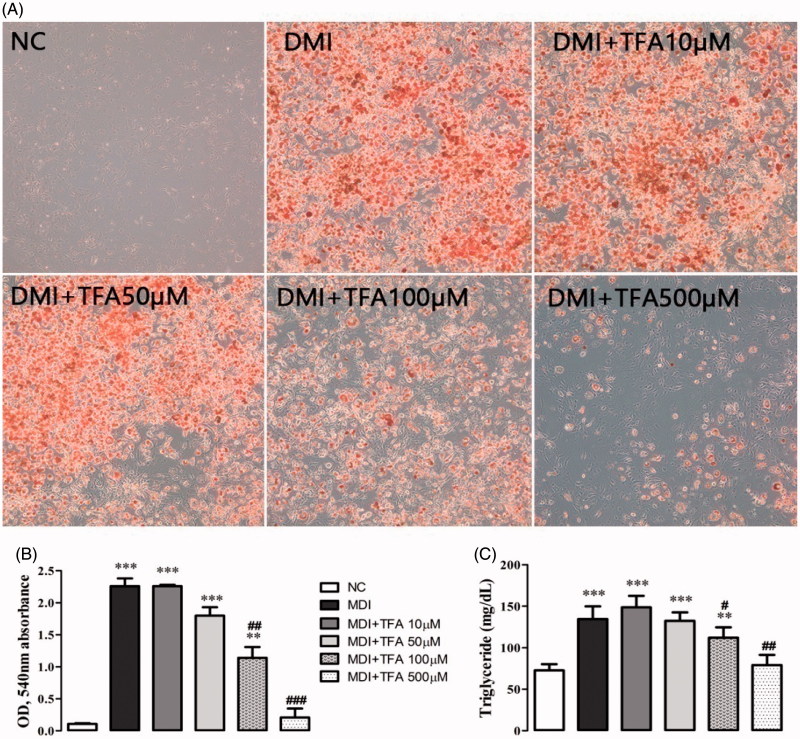
Effect of co-administration of DSP-derived *trans*-ferulic acid on lipid accumulation in 3T3-L1 cells. The data are reported as the mean ± SD (*n* = 10). *p < 0.05, **p < 0.01, ***p < 0.001, analyzed by parametric multiple comparison procedures, One-way ANOVA test. When the result of ANOVA was significant, and Dunnett’s multiple comparison test was applied versus the NC group; #*p* < 0.05; ##*p* < 0.01; and ###*p* < 0.001, versus DMI.

As shown in Figures 1 and 2, the purified compound DSP-EW-3 (compound 1) was identified as TFA. Intracellular toxicity was measured by MTT assay in 3T3-L1 cells after exposure to various concentrations of the purified TFA from DSP. As shown in Figure 5, there were no significant changes in cell viability. TFA reduced the cholesterol and triglyceride levels in differentiated adipocyte lysates in a dose-dependent manner (Figure 6). The absorbance values of Oil Red O staining decreased with the concentration of TFA in the DSP-treated group during adipocyte differentiation (Figure 6) from untreated fully differentiated cells. Triglyceride (85.09%, *p* < 0.001) was significantly increased in the DMI group compared to the NC group.

**Figure 7.**
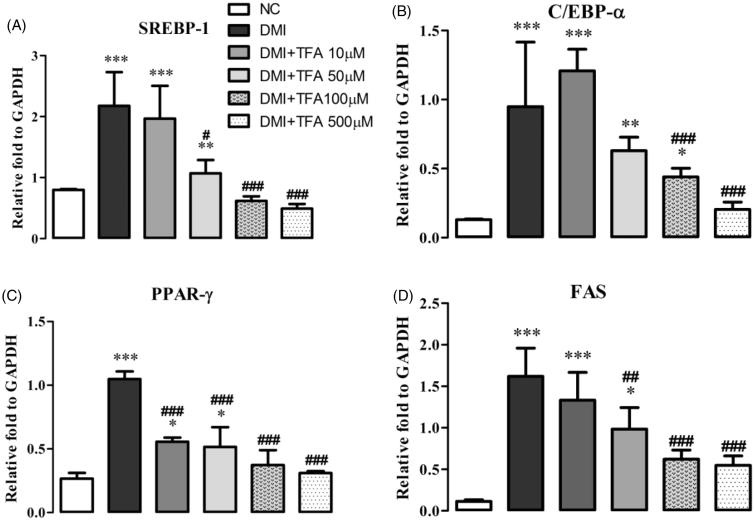
Effect of co-administration of DSP-derived *trans*-ferulic acid on gene/protein expression in 3T3-L1 cells. The data are reported as the mean ± SD (*n* = 10). *p < 0.05, **p < 0.01, ***p < 0.001, analyzed by parametric multiple comparison procedures, One-way ANOVA test. When the result of ANOVA was significant, and Dunnett’s multiple comparison test was applied versus the NC group; #*p* < 0.05; ##*p* < 0.01; and ###*p* < 0.001, versus DMI group.

The most therapeutically effective concentration was 500 mM, which reduced triglycerides by 41.25% (*p* < 0.001) compared to the DMI group. Co-treatment with TFA inhibited the overexpression of adipogenic-related genes SREBP1, PPARγ, C/EBPα and FAS (Figure 7) in comparison to the differentiated adipocytes in the DMI group. These results indicate that TFA may effectively inhibit adipocyte differentiation in 3T3-L1 cells.

## Discussion

The *in vivo* results demonstrate that DSP co-administration regulates weight gain and serum biochemical parameters (TC, TG, LDL, VLDL and AI) in HFD-induced obese rats. These results are similar to findings that NSP induces a reduction in body weight in animal models fed a high-salt and high-fat diet, which indicate that NSP could be used as a salt substitute to prevent weight gain (Hwang et al. 2007; Lee et al. 2012; Pichiah and Cha 2015).

Abdominal obesity is responsible for dyslipidemia characterized by increased serum cholesterol, LDL and triglycerides (Paccaud et al. 2000). Quantitative estimation of visceral obesity is thus important for evaluating the pathogenesis and for accurate prognosis. Visceral adiposity, TAF, SAF and VAF are all independently associated with health risks and metabolic syndrome (Goodpaster et al. 1997). However, VAF is more closely correlated with obesity associated pathologies and complications than either TAT or SAT (Judex et al. 2010). Therefore, it is important to stratify total adiposity into subcutaneous and visceral adiposity.

Non-invasive 3 D micro-CT imaging was performed to quantify adiposity (total and regional adipose volumes and fat infiltration). This method can readily discriminate between subcutaneous and visceral fat (Judex et al. 2010). Our results revealed that DSP was able to reduce TAF, VAF and SAF. This may have been due to the constituents of DSP, which include ferulic acid, caffeic acid, *p*-coumaric acid, isorhamnetin-3-β-d-glucoside and a high amount of dietary fibre (74.3%), which were also previously reported for NSP (Kong et al. 2009; Kong and Seo 2012; Panth et al. 2016). It was also proven that the desalting process has no negative impact on the chemical constituents of NSP.

Each component has a hypolipidemic effect individually. The anti-obesity and antioxidant activity of ferulic acid has been widely described in previous reports (Zhao and Moghadasian 2008; Yoon et al. 2013). It has also been reported that *p*-coumaric acid improves lipid metabolism via AMP-activated protein kinase in L6 skeletal muscle cells. In addition, multi-systemic beneficial effects of *p*-coumaric acid have also been reported, including anti-obesity and hypolipidemic action (Pei et al. 2016). Kong and Seo (2012) reported that NSP-derived glucopyranosides (Kong et al. 2012) and isohamnetin 3-*O*-β-d-glucopyranoside (Kong and Seo 2012) effectively suppressed adipogenic differentiation by down-regulation of PPARγ, C/EBPα, SREBP1 and adipocyte-specific proteins. Pichiah and Cha (2015) reported that the dietary fibre present in NSP exerts an anti-obesity effect. Furthermore, health benefits of dietary fibre including hypolipidemic and anti-obesity effects have been described (Mattes 2007; Anderson et al. 2009). Thus, it is noteworthy that co-treatment with DSP controlled obesity and improved the blood lipid profile, which was similar to that of the GE-treated group. These results might have been due to the chemical constituents and high dietary fibre content.

GE exerts anti-obesity effects due to its active component HCA (Marquez et al. 2012). To determine the underlying mechanism of the anti-obesity effects, DSP was analyzed by HPLC. TFA was identified as the most abundant active component. Panth et al. (2016) also identified TFA as a phenolic acid in NSP. Hypolipidimic (Senaphan et al. 2015; Naowaboot et al. 2016) and multi-systemic beneficial effects have been described for ferulic acid (Srinivasan et al. 2007). Hence, only TFA was selected for *in vitro* studies.

The anti-adipogenic effect of TFA on adipocyte differentiation was investigated in cultured 3T3-L1 adipocytes by measuring lipid accumulation and the expression of several transcription factors related to adipogenesis. Both adipocyte differentiation and lipid accumulation lead to obesity (Kang et al. 2010; Kim and Kong 2010). The results of Oil Red O staining and triglyceride measurements in cells showed that TFA significantly reduced triglyceride levels and the absorbance values of Oil Red O eluted solution in the cytoplasm of treated cells. This suggests that TFA inhibits adipogenesis during adipocyte differentiation by reducing lipid accumulation.

Adipocyte differentiation results from a series of programmed changes in specific genes or transcription factors involved in adipogenesis, such as PPARγ, C/EBPα, SREBP1 and FAS (Kim and Kong 2010; Ko et al. 2015; Guo et al. 2016). SREBP1 is associated with the production of an endogenous ligand that enhances PPARγ transcriptional activity and can increase the expression of several genes involved in fatty acid metabolism (Kang et al. 2010). Co-treatment with TFA markedly suppressed over expression of SREBP1 in this study. PPAR-γ and C/EBPα are found almost exclusively in adipose tissue and play a crucial role in adipocyte differentiation, indicating that these factors are linked to both the induction of adipose-specific genes and to the conversion of the mature adipose phenotype.

The addition of TFA down-regulated the activation of the key transcriptional regulators C/EBPα and PPARγ in 3T3-L1 adipocytes. The activation of FAS genes is involved in TG metabolism and inhibits the differentiation of early differentiating pre-adipocytes and lipogenesis in mature adipocytes (Song et al. 2013). The expression of FAS was also significantly lower in 3T3-L1 cells treated with TFA compared with terminally differentiated 3T3-L1 adipocyte control cells. This might have been due to the down-regulation of C/EBPα and PPARγ family members (Song et al. 2013).

## Conclusions

We have shown that the administration of DSP to rats with HFD-induced obesity reduces body weight gain, abdominal fat mass and serum lipid profiles. Furthermore, the biologically active component TFA inhibits adipogenesis and adipocyte differentiation by down-regulating the adipocyte-specific transcriptional regulators SREBP1, FAS, C/EBPα and PPARγ. The results show that DSP and its constituents may protect against HFD-induced obesity by inhibiting adipocyte differentiation and down-regulating adipogenic-related factors.
